# Pharmacological reprogramming of myeloid suppression in solid tumors: mechanisms, evidence standards for immune conversion, and biomarker-informed combinations

**DOI:** 10.3389/fphar.2026.1986842

**Published:** 2026-10-20

**Authors:** Nemanja Maletin, Nikola Denda, Milica Paut Kusturica, Aleksandar Rašković, Siniša Babović, Zoran Golušin

**Affiliations:** 1 Department of Pharmacology, Toxicology and Clinical Pharmacology, Faculty of Medicine, University of Novi Sad, Novi Sad, Serbia; 2 Clinic of Dermatovenereology, University Clinical Center of Vojvodina, Novi Sad, Serbia; 3 Clinic for Eye Diseases, University Clinical Center of Vojvodina, Novi Sad, Serbia

**Keywords:** CD40, CD47–SIRPα, CSF1R, MICAT, myeloid-derived suppressor cells, pharmacodynamic biomarkers, PI3Kγ, tumor microenvironment

## Abstract

Myeloid cells regulate immune exclusion, T-cell dysfunction, angiogenesis, stromal remodeling, and resistance to immune checkpoint blockade in solid tumors. Pharmacological development has moved from nonspecific depletion toward recruitment or survival blockade, intracellular state reprogramming, restoration of antigen presentation, and tumor-selective phagocytosis. Clinical experience nevertheless shows a recurrent gap between biological activity and attributable therapeutic immune conversion. We introduce the Myeloid Immune Conversion Audit Trail (MICAT), a fixed four-level extension of the pharmacological audit trail that grades intratumoral target engagement, conversion of the intended myeloid state, propagation through the immune ecosystem, and randomized clinical attribution. Each level is scored as not demonstrated, partial, or demonstrated. Applying MICAT across representative trials shows that most programs stop at systemic pharmacodynamics or partial lineage effects; few establish ecosystem propagation, and none yet completes the entire causal chain for an immunological switch in a solid-tumor carcinoma. The analysis identifies compensation and redundancy as the central biological obstacle: CCR2 blockade can recruit CXCR2-positive neutrophils, CSF1R inhibition can spare resistant SPP1-high macrophages, and CD40 or PI3Kγ activation can induce interferon-linked PD-L1 feedback. We therefore prioritize lineage-resolved paired tissue, preservation of cDC1 and cDC2 function, absolute cell quantification, orthogonal functional and spatial readouts, and randomized component-controlled trials. Disease context is decisive: tenosynovial giant-cell tumor establishes CSF1R dependence without proving immune conversion; macrophage-rich triple-negative breast cancer is a rational PI3Kγ test-bed; pancreatic ductal adenocarcinoma permits sequence-aware CD40 and stromal studies; and microsatellite-stable colorectal cancer is a stringent failure benchmark. CAR-macrophages, *in vivo* mRNA/lipid-nanoparticle reprogramming, myeloid engagers, and artificial-intelligence-assisted spatial scoring are mapped to the same evidence requirements. MICAT is intended as a reproducible tool for pharmacological go/no-go decisions rather than a claim that target modulation alone constitutes therapeutic immune conversion.

## 1 Introduction

Immune checkpoint inhibitors have established that endogenous antitumor immunity can produce durable cancer control, but a pre-existing or therapy-inducible tumor-reactive response must generally reach the lesion and retain sufficient function to be released from inhibition. Immune exclusion and dysfunction arise from interacting stromal, vascular, metabolic, and myeloid programs. A previous review characterized this broader continuum across tumor-microenvironment-directed interventions (Maletin, 2026). Here, we address the narrower pharmacological question of whether a myeloid-directed intervention can produce and causally validate intratumoral immune conversion.

Tumor-associated macrophages (TAMs), monocytes, neutrophils, myeloid-derived suppressor cells (MDSCs), and dendritic cells form an interdependent compartment rather than isolated drug targets. Pan-cancer single-cell analyses show recurrent myeloid programs alongside substantial tissue-, lesion-, and patient-specific variation (Cheng et al., 2021). MDSCs are likewise heterogeneous and are defined by suppressive function in addition to phenotype (Veglia et al., 2021). Spatial studies in pancreatic ductal adenocarcinoma (PDAC) demonstrate that location and cellular neighbors can be as consequential as marker profile (Liudahl et al., 2021). These observations challenge drug-development strategies based on bulk depletion or one surface marker.

Recent reviews have comprehensively described myeloid plasticity, lineage biology, clinical targets, antibody engineering, and cell- or RNA-based platforms (Sanseviero et al., 2026; Blanchard et al., 2025; Park et al., 2025). The novelty of the present review lies in its evaluative framework rather than in proposing a new catalogue of targets. We name this framework the Myeloid Immune Conversion Audit Trail (MICAT), apply a fixed scoring rubric trial by trial, and display the result as an agent-by-evidence matrix. MICAT makes the unproven links visible: a drug may reach its target yet fail to convert the intended lineage, or it may change that lineage without propagating through the immune ecosystem or improving outcome. The added ecosystem-propagation level is particularly important in myeloid pharmacology because depletion, redistribution, dendritic-cell injury, and compensatory recruitment can mimic or cancel a favorable proximal effect.

Because isolated changes in blood, cell abundance, cytokines, or CD8 density can occur without functional intratumoral conversion—and combination partners affect the same readouts—claims of reprogramming require a causal, tissue-based standard. This review focuses on CSF1/CSF1R, CCR2/CCR5 and CXCR1/2, PI3Kγ, CD40, and CD47–SIRPα, with additional assessment of Clever-1/STAB1, TREM2, and LILRB2. For each strategy, we distinguish intended mechanism, demonstrated pharmacodynamic effect, clinical activity, toxicity, biomarker needs, and the evidence still missing. The objective is to convert a descriptive field into a set of reproducible pharmacological decisions.

## 2 Review approach and interpretive principles

This targeted narrative review was developed through searches of PubMed, ClinicalTrials.gov, and publisher records, with emphasis on studies available through 17 September 2026. Search concepts combined solid tumor, tumor microenvironment, macrophage, monocyte, neutrophil, MDSC, dendritic cell, immune conversion, and the individual targets or agents discussed below. We prioritized peer-reviewed clinical trials with paired tissue or mechanistic analyses, randomized data, and recent publications from 2023 to 2026. Earlier studies were retained when they established a mechanism or remain necessary to interpret current development. Conference abstracts and registry-only results are explicitly identified and are not treated as equivalent to full peer-reviewed reports. Because the review was not designed as a systematic review, it does not provide an exhaustive intervention inventory or pooled efficacy estimate. Generative-AI assistance, disclosed in the Acknowledgments, did not independently select studies or assign evidence levels.

Three interpretive rules were applied. First, changes in blood were considered supportive pharmacodynamic evidence but not a substitute for intratumoral measurement. Second, immune-cell abundance and state were evaluated separately. Third, single-arm response rates were interpreted against uncertainty from patient selection, treatment backbone, disease biology, and small sample size. A promising signal was therefore distinguished from evidence that the experimental drug contributed clinical benefit.

## 3 What constitutes a pharmacological myeloid immune switch?

### 3.1 Myeloid states are continuous, contextual, and reversible

The M1/M2 framework remains useful as historical shorthand but is inadequate as a pharmacodynamic endpoint. Human TAMs frequently co-express inflammatory, scavenging, angiogenic, lipid-handling, matrix-remodeling, and antigen-presentation programs. SPP1-high macrophages are associated with fibrotic and immune-excluding niches, whereas CXCL9-high macrophages track with interferon signaling and productive lymphocyte interactions (Bill et al., 2023). These programs are neither mutually exclusive nor stable across treatment, metastatic organs, and time. Monocyte-derived and tissue-resident macrophages may also respond differently to the same drug.

For MDSCs, phenotype is particularly vulnerable to misclassification. Polymorphonuclear MDSCs overlap phenotypically with neutrophils, and monocytic MDSCs overlap with conventional monocytes. Suppression assays, density-gradient methods, cryopreservation, and marker panels introduce additional variation (Veglia et al., 2021). Trials should predefine the population being targeted, specify specimen handling, and report whether a decline reflects altered trafficking, depletion, maturation, or loss of suppressive function.

Dendritic-cell heterogeneity is equally consequential but is often hidden inside broad CD11c- or HLA-DR-positive gates. Conventional type 1 dendritic cells (cDC1s) specialize in uptake and cross-presentation of tumor antigen to CD8 T cells, produce IL-12 and CXCL9/CXCL10, and connect natural-killer-cell signals with cytotoxic immunity. Conventional type 2 dendritic cells (cDC2s) are more heterogeneous and are central to CD4 T-cell priming, helper polarization, and coordination of humoral and cytotoxic responses; inflammatory DC3 and monocyte-derived DC states further blur lineage boundaries (Wculek et al., 2020). A favorable macrophage change cannot satisfy ecosystem propagation if cDC1 cross-presentation or productive cDC2 help is lost.

Myeloid-directed drugs can influence these DC compartments through intended biology or off-target pharmacology. CD40 agonists may activate both cDC1 and cDC2, but their effect depends on receptor clustering, tissue localization, and antigen availability. In contrast, the multikinase activity of pexidartinib against FLT3 and c-KIT impaired DC differentiation and reduced DC subsets in MEDIPLEX, providing a clinically observed example in which a nominal macrophage intervention damaged the antigen-presenting bridge needed for checkpoint therapy (Voissière et al., 2024). This finding should not be generalized to all CSF1R inhibitors, but it requires future trials to enumerate cDC1 and cDC2 separately, assess cross-presentation or a validated functional surrogate, and distinguish DC loss from maturation. CD47–SIRPα and LILRB2 agents also require lineage-resolved assessment because their targets can affect antigen uptake and inhibitory signaling beyond macrophages.

### 3.2 Four pharmacological operations

Myeloid-directed agents perform four broad operations (Figure 1; Table 1). Depletion or survival blockade removes cells that depend on a target such as CSF1R. Recruitment blockade limits the influx of CCR2-positive monocytes or CXCR2-positive neutrophilic cells. State reprogramming changes intracellular signaling without requiring cell loss, exemplified by PI3Kγ inhibition. Functional activation restores antigen presentation, co-stimulation, or phagocytosis, as pursued through CD40 agonism and CD47–SIRPα blockade. These operations are not interchangeable. Depletion may open physical space but remove useful antigen-presenting subsets. Recruitment blockade may work only while dosing is maintained and can trigger compensation through another chemokine axis. Reprogramming requires proof that the same lineage changed state. Functional activation requires an antigen source and a therapeutic window that avoids systemic inflammation or normal-cell phagocytosis.

**FIGURE 1:**
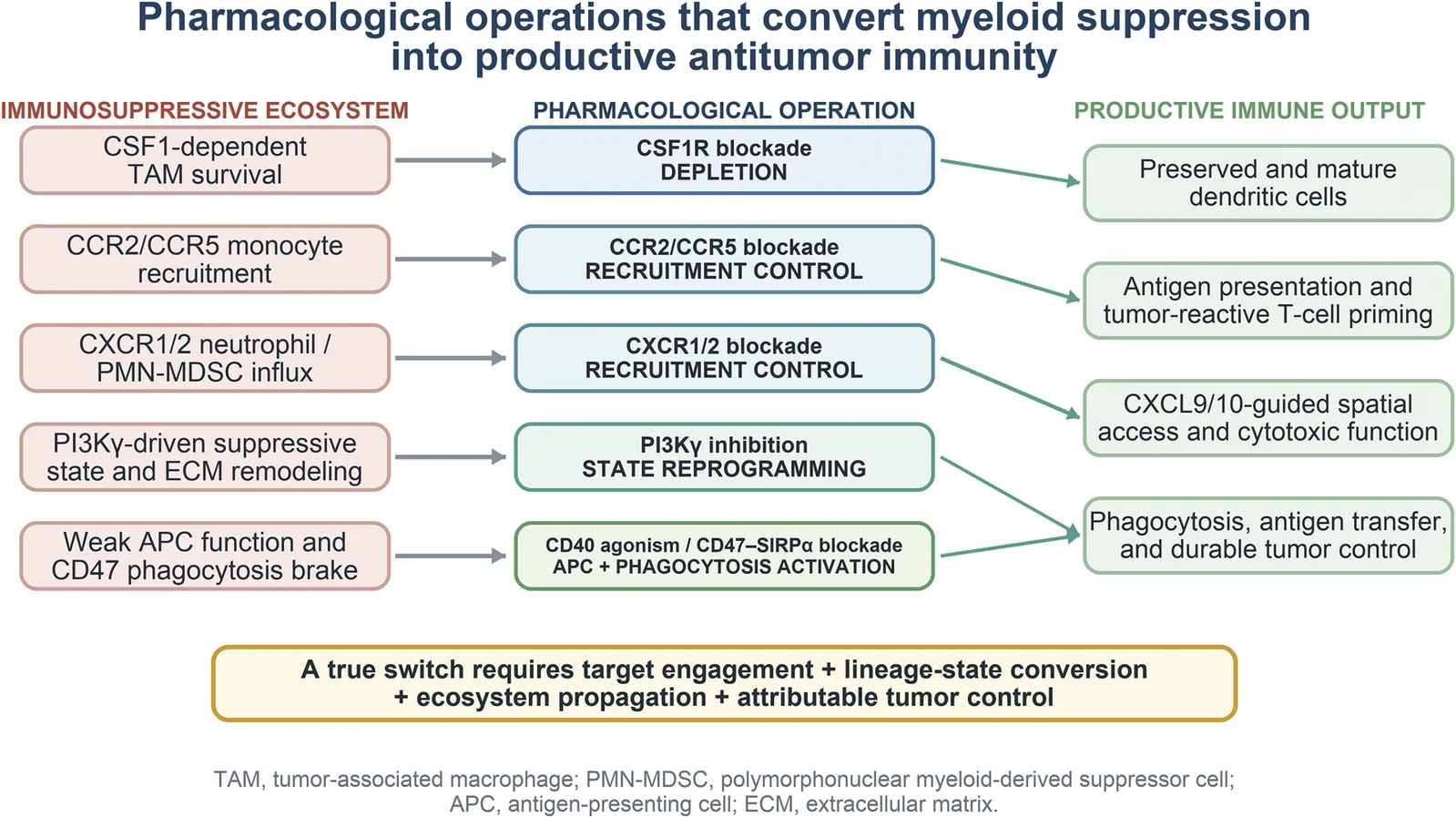
Pharmacological operations that can convert myeloid suppression into productive antitumor immunity. Suppressive myeloid ecosystems are maintained through survival signals, continuous recruitment, intracellular state programs, weak antigen presentation, inhibitory phagocytosis checkpoints, and stromal organization. CSF1R blockade removes survival-dependent cells; CCR2/CCR5 and CXCR1/2 antagonists reduce recruitment; PI3Kγ inhibitors reprogram intracellular signaling; CD40 agonists license antigen-presenting cells; and CD47–SIRPα blockade restores phagocytosis when a tumor-selective pro-phagocytic signal is present. A productive switch requires preserved cDC1/cDC2 function, spatial access, tumor-reactive T-cell activation, and tumor control. TAM, tumor-associated macrophage; MDSC, myeloid-derived suppressor cell; DC, dendritic cell; APC, antigen-presenting cell; ECM, extracellular matrix.

**TABLE 1:** Mechanistic classes of myeloid-directed pharmacology.

Target/operation	Representative agents	Intended immune conversion	Minimum tissue evidence	Main liabilities
CSF1/CSF1R; survival/depletion	Pexidartinib, emactuzumab, AMG 820	Remove CSF1-dependent suppressive TAMs and permit T-cell access	Loss of specified TAM state with preserved cDC1/cDC2 and cross-presentation, plus increased effector function	Edema, enzyme elevations, hepatotoxicity; loss of beneficial macrophage/DC populations; resistant SPP1-high or alternative survival states
CCR2/CCR5; recruitment blockade	PF-04136309, BMS-813160	Reduce influx of monocytic suppressor cells	Decline in tumor—not only blood—CCR2/CCR5-dependent cells plus reduced suppressive function	Redundant chemokines, CXCR2-positive neutrophil substitution, rebound recruitment, partner-dependent pulmonary toxicity
CXCR1/2; recruitment blockade	Navarixin, reparixin, AZD5069	Reduce neutrophil/PMN-MDSC recruitment and relieve T-cell exclusion	Reduced intratumoral suppressive neutrophilic state with preserved host defense	Neutropenia, infection, compensatory macrophage recruitment; weak activity in unselected tumors
PI3Kγ; intracellular reprogramming	Eganelisib	Shift macrophages from SPP1/ECM-remodeling toward CXCL9/10, HLA, and interferon programs	Within-patient state-score change with stable absolute density or explicit accounting for depletion	Transaminitis, rash; interferon/PD-L1 feedback; causal attribution in multi-agent regimens remains unproven
CD40; APC activation	Selicrelumab, sotigalimab, mitazalimab, MP0317, RO7300490	License cDC1/cDC2, enhance cross-priming, remodel macrophages/stroma	Tumor-localized pathway activation, mature DC subsets, new or expanded tumor-reactive TCR clones	Infusion reactions, CRS, thrombocytopenia, liver injury; activation geometry, schedule, antigen source, and PD-L1 feedback
CD47–SIRPα; phagocytosis checkpoint	Magrolimab, evorpacept, SL-172154	Restore macrophage phagocytosis and antigen transfer	Tumor-cell phagocytosis with retained tumor antigen/opsonization and downstream T-cell activation	Anemia, thrombocytopenia, transfusion interference, CRS/HLH; normal-cell antigen sink
Clever-1/STAB1, TREM2, LILRB2; emerging checkpoints	Bexmarilimab, PY314, MK-4830, IO-108	Remove or reprogram suppressive macrophage subsets	Target-defined lineage change plus evidence the population is causally required	Early-phase signals, no validated selection assay, peripheral rather than tumor occupancy, lineage compensation

Abbreviations: APC, antigen-presenting cell; CRS, cytokine-release syndrome; DC, dendritic cell; ECM, extracellular matrix; HLA, human leukocyte antigen; HLH, hemophagocytic lymphohistiocytosis; PMN-MDSC, polymorphonuclear myeloid-derived suppressor cell; TAM, tumor-associated macrophage; TCR, T-cell receptor.

### 3.3 The myeloid immune conversion audit trail

The Pharmacological Audit Trail (PhAT) linked patient selection, exposure, target engagement, pathway modulation, biological effect, intermediate response, and tumor outcome into a continuous drug-development logic (Banerji and Workman, 2016; Yap et al., 2010). MICAT retains that lineage but adapts it to the multicellular and compensatory nature of myeloid pharmacology. Its distinctive addition is an explicit ecosystem-propagation level between target-lineage modulation and clinical outcome. This prevents a change in the targeted cell from being credited as immune conversion when dendritic cells, T cells, stromal access, or an alternative suppressive lineage remain unchanged or worsen.

Representative clinical studies and the evidence they establish are summarized in Table 2. MICAT contains four linked levels (Table 3). Level 1 is intratumoral target engagement, demonstrated by tumor exposure, receptor occupancy or colocalization, proximal signaling, or a time- and dose-linked tissue pharmacodynamic effect. Level 2 is targeted myeloid-state conversion, shown by a prespecified lineage-specific functional or state change while accounting for depletion and redistribution. Level 3 is immune-ecosystem propagation, requiring concordant downstream evidence in at least two orthogonal domains, including antigen-presenting-cell function, adaptive-effector function, clonal change, or spatial organization. Level 4 is randomized clinical attribution, in which a component-controlled comparison shows benefit from the experimental agent. When conversion biomarkers are available, their measurement before outcome and formal interaction or mediation analysis determine whether the clinical effect is linked to immune conversion.

**TABLE 2:** Selected clinical studies and what they establish.

Strategy/study	Design and disease	Key pharmacodynamic observation	Clinical result	Interpretation for immune conversion
Pexidartinib ENLIVEN (Tap et al., 2019)	Randomized phase III, TGCT	CSF1R-pathway-dependent disease; no immune-conversion package	Week-25 response 39% versus 0%	Randomized target-dependent efficacy; validates Level 4 tumor control but not Levels 2–3 immune conversion in carcinoma
Pexidartinib + durvalumab MEDIPLEX (Voissière et al., 2024)	Phase I, colorectal cancer/PDAC, n = 47	Systemic CSF1R PD; off-target FLT3/c-KIT inhibition reduced DCs and IFN-λ function	One partial response in MSI-high colorectal cancer	Kinase selectivity and DC preservation are essential; tissue conversion and attribution absent
Emactuzumab + atezolizumab (Gomez-Roca et al., 2022)	Phase Ib, advanced solid tumors, n = 221	TAM reduction and activated-CD8 association	ORR 5.6%–12.5% across cohorts	Tissue PD did not show checkpoint-resistance reversal
BMS-813160 combinations (Le et al., 2026; Grierson et al., 2025)	Open-label phase Ib/II, PDAC/colorectal cancer	Recruitment and inflammatory biomarkers; neoadjuvant tissue showed fewer monocytes/macrophages	No clear improvement across cohort comparisons	Biologically active; compensation and component contribution unresolved
Navarixin + pembrolizumab (Armstrong et al., 2024)	Phase II, CRPC/MSS colorectal cancer/NSCLC, n = 105	Peripheral neutrophil reduction	Three partial responses; closed for futility	Systemic PD is insufficient; tumor selection and tissue proof are required
MARIO-3 eganelisib triplet (O'Connell et al., 2024)	Single-arm phase II, first-line metastatic TNBC, n = 62	Increased HLA/CXCL9/10 and reduced SPP1/ECM in paired biopsies	Single-arm efficacy; no component-controlled estimate	Strong Levels 2–3 regimen signal; direct Level 1 and Level 4 attribution missing
PRINCE (Padrón et al., 2022)	Randomized phase II, first-line metastatic PDAC	Exploratory CD4/DC/APC features associated with sotigalimab benefit	Historical 1-year OS benchmark met only by nivolumab/chemotherapy cohort	Neither sotigalimab arm met its benchmark; no attributable CD40 benefit
OPTIMIZE-1 (Van Laethem et al., 2024; Van Laethem et al., 2025)	Single-arm phase Ib/II, first-line metastatic PDAC	Dynamic peripheral immune and ctKRAS changes	ORR 42.1%; median OS 14.9 months in update	Promising regimen; intratumoral conversion and component contribution unresolved
MP0317/RO7300490 (Steeghs et al., 2026; Melero et al., 2026)	First-in-human FAP-targeted CD40 studies, n = 46/n = 80	Tumor localization, CD40 activation, partial DC maturation	One unconfirmed response/no objective responses	Direct evidence that delivery and partial activation do not ensure tumor control
Magrolimab HNSCC (Colevas et al., 2026)	Randomized/nonrandomized phase II cohorts	No complete tissue conversion chain	No first-line PFS benefit; more grade 3–4 toxicity	No favorable efficacy–toxicity balance; early closure limits precision
ELEVATE CRC (Fakih et al., 2026)	Randomized phase II, previously treated metastatic CRC, n = 67	No complete tissue conversion chain	Median PFS 6.2 versus 6.7 months; more grade ≥3 events	Randomized evidence did not show added disease control
ASPEN-06 evorpacept (Shitara et al., 2025)	Randomized phase II, HER2-positive gastric/GEJ cancer; conference report	Fc-inactive CD47 blockade plus HER2 opsonization	Exploratory higher activity with fresh-biopsy HER2 retention	Biomarker-matched signal; full report, paired tissue, and confirmatory attribution required
SL-172154 (Lakhani et al., 2025)	Phase I, platinum-resistant ovarian cancer, n = 27	Peripheral engagement; paired-tumor macrophage/CD8 shifts	No objective responses	Partial Levels 1–3 did not yield tumor control
Bexmarilimab MATINS (Rannikko et al., 2023)	Phase I/II, advanced solid tumors	IFNγ, TCR, and macrophage activation in small paired subset	Disease control in selected histologies	Early reprogramming evidence; randomized validation and locked biomarker needed
PY314 + pembrolizumab (Beckermann et al., 2024)	Phase Ib, checkpoint-refractory RCC, n = 17	Limited evidence of TREM2-lineage depletion in tumor	One partial response; median PFS 1.4 months	Feasible dosing; target dependence, compensation, and efficacy unsupported
MK-4830/IO-108 (Siu et al., 2022; Taylor et al., 2024)	Phase I LILRB2 programs, mixed solid tumors	Dose-related or peripheral occupancy; exploratory inflammatory signatures	Early responses in uncontrolled cohorts	Feasibility shown; intratumoral engagement, lineage conversion, and attribution missing

Abbreviations: APC, antigen-presenting cell; CRC, colorectal cancer; CRPC, castration-resistant prostate cancer; ctKRAS, circulating KRAS-mutant allele frequency; DC, dendritic cell; ECM, extracellular matrix; FAP, fibroblast activation protein; GEJ, gastroesophageal junction; HLA, human leukocyte antigen; HNSCC, head and neck squamous-cell carcinoma; IFN, interferon; MSS, microsatellite stable; NSCLC, non-small-cell lung cancer; ORR, objective response rate; OS, overall survival; PD, pharmacodynamic; PDAC, pancreatic ductal adenocarcinoma; PFS, progression-free survival; TAM, tumor-associated macrophage; TGCT, tenosynovial giant-cell tumor; RCC, renal-cell carcinoma; TNBC, triple-negative breast cancer; TCR, T-cell receptor.

**TABLE 3:** Fixed MICAT scoring rubric.

Level	Score 0: not demonstrated or negative	Score 1: partial or indirect	Score 2: demonstrated	Guardrail
1. Intratumoral target engagement	No tumor evidence, or negative tumor assay	Systemic exposure/occupancy, imaging, or indirect tissue effect without direct tumor engagement and exposure–response	Direct intratumoral engagement plus a time- or dose-linked proximal PD effect	Blood occupancy or cytokines alone cannot score 2
2. Targeted myeloid-state conversion	No target-lineage conversion	Abundance or signature change confounded by depletion/redistribution, or lacking orthogonal confirmation	Prespecified lineage-specific functional/state change with orthogonal confirmation and absolute abundance accounted for	A favorable fraction created by selective depletion cannot score 2
3. Immune-ecosystem propagation	No downstream ecosystem evidence	One downstream domain or an ambiguous change	Concordant changes in at least two orthogonal domains, including APC/adaptive function, clonality, or spatial organization	CD8 density without function or persistent counter-suppression cannot score 2
4. Randomized clinical attribution	No randomized evidence or randomized negative result	Single-arm/historical signal, or exploratory randomized result without a robust component estimate	Component-controlled randomized benefit attributable to the agent; when conversion is measured, it precedes outcome and is tested by interaction or mediation analysis	A response signature found after unblinding cannot upgrade the score

Abbreviations: APC, antigen-presenting cell; MICAT, myeloid immune conversion audit trail; PD, pharmacodynamic.

Each level receives a fixed score: 0 when evidence is absent or negative, one when it is incomplete or indirect, and 2 when the criterion is demonstrated according to Table 3. The total ranges from 0 to 8 but measures completeness of causal evidence, not drug potency, commercial value, or probability of future success. Levels are not interchangeable: randomized efficacy in a directly target-dependent neoplasm can produce a Level 4 score without proving Levels 2–3 immune conversion, while extensive tissue biology in a single-arm combination cannot earn Level 4. Negative randomized trials receive 0 at Level 4 because they provide strong evidence that attributable benefit was not demonstrated.

Figure 2 applies this rubric to the clinical studies reviewed here. Two reviewers should ideally score each study independently from a prespecified evidence table and resolve disagreements by consensus. The matrix shows the dominant field-wide pattern: systemic or partial target pharmacology is common, lineage conversion is less consistent, ecosystem propagation is uncommon, and no carcinoma study completes all four levels. Accordingly, Level 1 establishes pharmacological activity; Levels 1–3 together establish biological conversion; and only completion of all four levels supports a clinically validated myeloid immune switch.

**FIGURE 2:**
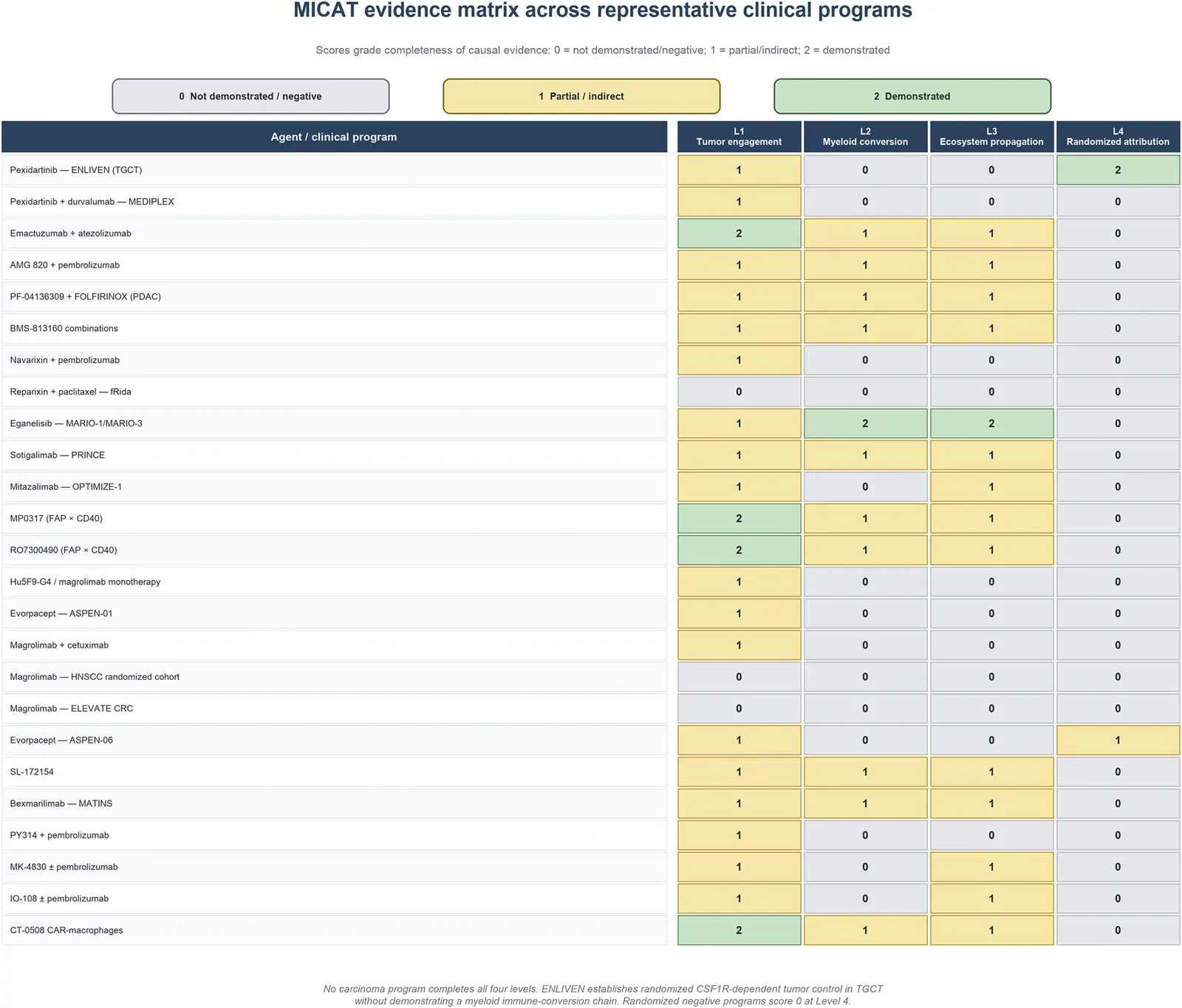
MICAT evidence matrix across representative clinical programs. The criteria in Table 3 are encoded as grey for score 0, amber for score 1, and green for score 2. A randomized negative study receives 0 at Level 4 because attributable benefit was not shown. The matrix grades evidence completeness rather than efficacy or future potential and shows that most programs stop at Levels 1–2, with isolated Level 3 signals and no carcinoma program completing all four levels. TGCT demonstrates randomized CSF1R-dependent tumor control without establishing an immune-conversion chain. CAR-M, chimeric antigen receptor macrophage; HNSCC, head and neck squamous-cell carcinoma; TGCT, tenosynovial giant-cell tumor; GEJ, gastroesophageal junction; TNBC, triple-negative breast cancer; RCC, renal-cell carcinoma; PDAC, pancreatic ductal adenocarcinoma.

## 4 Pharmacological strategies

### 4.1 CSF1/CSF1R blockade: depletion is measurable, selectivity is decisive

CSF1R signaling supports the survival, differentiation, and maintenance of many macrophage populations. Preclinical studies showed that CSF1R inhibition can alter macrophage polarization and suppress glioma progression (Pyonteck et al., 2013), while CSF1/CSF1R blockade in PDAC models reprogrammed infiltrating macrophages and improved checkpoint therapy (Zhu et al., 2014). These results supplied a strong rationale for small-molecule inhibitors and monoclonal antibodies, but translation into unselected solid tumors has been limited.

The target is clinically valid in a distinctly CSF1-dependent disease. In the randomized ENLIVEN trial of advanced tenosynovial giant-cell tumor, pexidartinib produced a week-25 response in 39% of patients versus none with placebo, while mixed or cholestatic hepatotoxicity prompted early closure of enrollment (Tap et al., 2019). This direct antitumor effect in a CSF1-driven neoplasm should not be interpreted as proof of immune conversion in carcinomas; it instead shows how pathway dependence can determine target-level efficacy.

The modern clinical lesson is that a nominally macrophage-directed small molecule can damage adjacent immune functions when its kinase selectivity is insufficient. In the phase I MEDIPLEX trial, pexidartinib plus durvalumab produced systemic CSF1R pharmacodynamic effects in advanced colorectal and pancreatic cancers, but only one of 47 patients achieved a partial response; the tumor was MSI-high colorectal cancer. Pexidartinib also inhibited FLT3 and c-KIT, impaired FLT3-dependent dendritic-cell differentiation, reduced dendritic-cell subsets, and weakened interferon-λ-related function, providing a mechanistic explanation for antagonism rather than synergy with PD-L1 blockade (Voissière et al., 2024). This off-target effect should not be generalized to selective CSF1R blockade; it demonstrates that target selectivity and preservation of cross-presentation can determine whether a macrophage-directed strategy helps or harms the intended immune response.

Antibody approaches have produced the same target-engagement/efficacy separation. Emactuzumab plus atezolizumab reduced CSF1R-dependent populations and generated objective response rates of 5.6%–12.5% across evaluable cohorts, without evidence that the combination overcame prior checkpoint resistance (Gomez-Roca et al., 2022). AMG 820 plus pembrolizumab increased CSF1 and IL-34, reduced selected circulating monocytes, and increased several intratumoral immune markers, but immune-related partial responses occurred in only 3% of 116 patients and no expansion cohort crossed its activity threshold (Razak et al., 2020). Facial or periorbital edema, transaminase elevations, fatigue, and rash recur across the class; pexidartinib additionally requires attention to serious mixed or cholestatic hepatotoxicity.

These data do not invalidate CSF1R as a target. They narrow the conditions under which it is likely to work. CSF1-driven tumors with unusually strong pathway dependence are biologically different from carcinomas in which multiple macrophage survival signals coexist. Future carcinoma trials should select the targeted TAM state, document its depletion in tissue, measure preservation of conventional dendritic cells and cross-presentation, and compare pulsatile with continuous schedules. Serum CSF1/IL-34 and circulating monocyte changes should be treated as exposure markers rather than predictive biomarkers.

### 4.2 CCR2/CCR5 and CXCR1/2 blockade: controlling recruitment in a redundant network

Blocking chemokine receptors aims to prevent suppressive precursors from continuously replenishing the TME. CCR2 is central to monocyte mobilization and recruitment, CCR5 participates in myeloid and lymphoid trafficking, and CXCR1/2 mediate recruitment of neutrophils and polymorphonuclear MDSC-like cells. This strategy is attractive because it can spare resident cells, but it is vulnerable to redundant ligands, alternative receptors, organ-specific trafficking, and rapid rebound after drug withdrawal.

The dual CCR2/5 antagonist BMS-813160 illustrates both biological activity and uncertain component contribution. In a 2026 phase 1b/2 study, first-line PDAC response rates were 37% with BMS-813160, gemcitabine/nab-paclitaxel, and nivolumab; 26% with BMS-813160 plus chemotherapy; and 28% with chemotherapy alone. Twenty-four-week progression-free survival was 56%, 56%, and 50%, respectively. In second-line colorectal cancer, neither experimental regimen clearly improved activity over FOLFIRI (Le et al., 2026). A neoadjuvant PDAC study showed reduced intratumoral monocytes/macrophages and increased T-cell proliferation and effector programs, but included 32 experimental patients and only eight controls, limiting efficacy inference (Grierson et al., 2025). These studies support target activity, not a validated clinical switch.

Earlier CCR2 studies explain why backbone and context matter. PF-04136309 plus FOLFIRINOX in borderline-resectable or locally advanced PDAC reduced circulating and tumor monocytes and produced a strong response signal in a small nonrandomized study (Nywening et al., 2016). The same antagonist with gemcitabine/nab-paclitaxel in metastatic PDAC produced no convincing added efficacy and was associated with pulmonary toxicity in 24% of patients, including grade 4 hypoxia (Noel et al., 2020). A trafficking target can therefore interact differently with cytotoxic backbones, disease stage, and organ reserve.

CXCR2 inhibition has also produced mixed results. Navarixin plus pembrolizumab generated little activity across castration-resistant prostate cancer, microsatellite-stable colorectal cancer, and non-small-cell lung cancer, and the trial closed for futility (Armstrong et al., 2024). In a mechanistically enriched prostate-cancer study, AZD5069 plus enzalutamide linked senescence-associated CXCR2 ligands to myeloid chemotaxis; five of 21 response-evaluable patients met a composite endpoint comprising RECIST tumor reduction, PSA50, or circulating-tumor-cell conversion, although only 13 had measurable disease (Gu et al., 2023). The randomized fRida trial of reparixin plus paclitaxel in metastatic triple-negative breast cancer was negative, with nearly identical median progression-free survival between arms (Goldstein et al., 2021).

The appropriate biomarker is unlikely to be receptor expression alone. Trials should integrate baseline neutrophil-to-lymphocyte ratio, circulating CCR2-positive monocytes, a ligand or senescence-associated secretory-phenotype signature measured in fresh tumor tissue, and early confirmation that the intended population was removed from the lesion rather than redistributed from blood. Neutropenia can indicate CXCR2 pharmacology but is simultaneously a safety risk. Multi-axis or adaptive recruitment blockade may be required, but broader suppression increases infection and marrow risk.

### 4.3 PI3Kγ inhibition: reprogramming without mandatory depletion

PI3Kγ is enriched in myeloid cells and integrates receptor signals that favor immune suppression. Foundational studies showed that PI3Kγ operates as a macrophage molecular switch between an AKT/mTOR–C/EBPβ-associated suppressive program and NF-κB-associated inflammatory signaling (Kaneda et al., 2016), and that its inhibition can overcome resistance to checkpoint blockade in preclinical models (De Henau et al., 2016). In principle, this strategy preserves macrophages while changing their function, which may retain antigen presentation and tissue homeostasis better than broad depletion.

Eganelisib is the most clinically developed PI3Kγ inhibitor. The phase one/1b MARIO-1 trial showed transaminitis and rash alongside activity signals in monotherapy and nivolumab cohorts, but its nonrandomized design could not establish additive efficacy (Hong et al., 2023). In frontline metastatic triple-negative breast cancer, the MARIO-3 triplet of eganelisib, atezolizumab, and nab-paclitaxel increased MHC class I/II and CXCL9/CXCL10 programs, reduced SPP1, metalloprotease, and extracellular-matrix programs, and shifted the intratumoral CXCL9:SPP1 balance (O'Connell et al., 2024). Paired biopsies were collected from 29 patients, while the reported digital-spatial and multiplex-imaging subsets were smaller (n = 12 and n = 11, respectively).

The regimen-level changes are consistent with Levels 2–3, but agent-specific intratumoral PI3Kγ engagement was not shown. Atezolizumab and nab-paclitaxel can independently alter the same programs, and the progression-free-survival association was exploratory. A decisive study should randomize the same backbone with or without PI3Kγ inhibition, lock the conversion score before unblinding, and select dose by tissue pharmacodynamic plateau and reversible hepatic toxicity.

### 4.4 CD40 agonism: restoring antigen presentation and coordinating sequence

CD40 agonism can license dendritic cells, increase co-stimulation and cross-priming, and alter macrophage-mediated stromal remodeling. Agents are not pharmacologically interchangeable: epitope, valency, receptor clustering, FcγRIIB dependence or Fc engineering, tumor anchoring, dose, and schedule shape agonism and toxicity (Beatty et al., 2011; Byrn et al., 2021; Padrón et al., 2022; Van Laethem et al., 2024; Van Laethem et al., 2025; Weiss et al., 2024; Steeghs et al., 2026; Melero et al., 2026). In PDAC, early and neoadjuvant studies showed stromal effects, greater dendritic-cell maturation, and more T-cell-enriched tumors relative to historical specimens (Beatty et al., 2011; Byrn et al., 2021).

Clinical results show that schedule and partner therapy matter. In the randomized phase II PRINCE trial, the prespecified 1-year overall-survival benchmark was met by nivolumab plus chemotherapy, but neither sotigalimab-containing cohort met that historical benchmark; the design was not powered to establish arm-to-arm superiority or inferiority. Exploratory CD4, dendritic-cell, and antigen-presenting-cell features were associated with benefit in the sotigalimab cohort (Padrón et al., 2022). OPTIMIZE-1 used an initial mitazalimab lead-in 1 week before mFOLFIRINOX and reported encouraging response and survival estimates, with dynamic circulating immune changes and circulating KRAS-mutant allele frequency (ctKRAS) associated with outcome (Van Laethem et al., 2024; Van Laethem et al., 2025). The single-arm design leaves the mitazalimab contribution unresolved.

Sotigalimab plus nivolumab produced durable responses in a subset of patients with melanoma progressing on anti-PD-1 therapy (Weiss et al., 2024). Tumor-localized agonists have clarified the difference between delivery and therapeutic conversion: MP0317 and RO7300490 achieved localization and partial pathway activation, yet objective tumor regression was absent or exceptional (Steeghs et al., 2026; Melero et al., 2026).

Future studies should adapt sequence to each agonist’s receptor-clustering pharmacology, antigen source, and partner therapy. Baseline and on-treatment dendritic-cell competence may be more informative than tumor CD40 expression alone.

### 4.5 CD47–SIRPα blockade: phagocytosis requires tumor selectivity

The CD47–SIRPα axis transmits a “do not eat me” signal that restrains macrophage phagocytosis. Blocking the axis is conceptually direct but pharmacologically difficult because CD47 is widely expressed on normal cells, including erythrocytes and platelets. Active-Fc anti-CD47 antibodies can create an antigen sink, acute anemia, thrombocytopenia, hemagglutination, transfusion-testing interference, and infusion reactions. The first-in-human Hu5F9-G4 study established a priming-dose strategy that reduced acute erythrocyte toxicity, but monotherapy activity was limited (Sikic et al., 2019).

Two design solutions have emerged. The first uses an Fc-inactive blocker together with a separate tumor-opsonizing antibody. In the small ASPEN-01 expansion cohorts, evorpacept combinations produced responses in 4/20 patients with head and neck cancer, 1/20 with non-small-cell lung cancer, and 4/19 with HER2-positive gastric/gastroesophageal cancer, while monotherapy produced none (Lakhani et al., 2021). The second retains an active phagocytic antibody while controlling dose and partner selection; magrolimab plus cetuximab showed limited, KRAS-dependent activity and clinically relevant anemia (Eng et al., 2025).

Later studies underscore the need for randomized and biomarker-defined development. In first-line head and neck squamous-cell carcinoma, adding magrolimab to pembrolizumab and chemotherapy did not improve progression-free survival and increased grade 3–4 adverse events; early closure limits precision (Colevas et al., 2026). ELEVATE CRC was also closed after 67 of the planned 117 patients had been randomized: median progression-free survival was 6.2 months with magrolimab plus bevacizumab-FOLFIRI and 6.7 months with bevacizumab-FOLFIRI alone, while grade 3 or higher treatment-emergent adverse events occurred in 72.7% and 52.4%, respectively (Fakih et al., 2026). By contrast, the conference-reported ASPEN-06 study generated a hypothesis that fresh-biopsy confirmation of retained HER2 may enrich activity for evorpacept with HER2-directed therapy, but its intention-to-treat historical comparison was nonsignificant and the between-arm subgroup comparison was exploratory (Shitara et al., 2025).

Excessive innate and adaptive activation can also be dangerous. The trial of evorpacept, cetuximab, and pembrolizumab in refractory microsatellite-stable colorectal cancer was stopped early after two treatment-related grade 5 events: one case each of hemophagocytic lymphohistiocytosis and cytokine-release syndrome (Lentz et al., 2025). SL-172154, a bifunctional CD47 inhibitor/CD40 agonist, achieved near-complete peripheral target engagement, cytokine induction, a tumor macrophage-state shift, and greater CD8 infiltration, but no objective responses in platinum-resistant ovarian cancer (Lakhani et al., 2025). These findings separate systemic pharmacology and partial tissue remodeling from tumor control.

The most actionable current biomarker is preservation of the target used for opsonization, such as HER2 confirmed near treatment by fresh tissue or circulating tumor DNA. Other candidates include tumor CD47 density, Fcγ receptor-competent macrophages, pro-phagocytic calreticulin or antibody coating, and SIRPα genotype for allele-selective agents. Every study should monitor hemolysis markers, transfusion requirements, platelet counts, cytokines, and macrophage function.

### 4.6 Emerging myeloid checkpoints: promising biology, early clinical validation

The early agents in this section remain dose-finding programs, so safety, schedule, target accessibility, and biomarker performance are part of the mechanistic result rather than secondary details (Table 4). Clever-1, also known as stabilin-1, marks scavenging and immunoregulatory macrophage functions. In MATINS, intravenous bexmarilimab was escalated from 0.1 to 10 mg/kg every 3 weeks. No dose-limiting toxicity was observed; half of 138 treated patients had a treatment-related adverse event, 8.7% had grade 3–4 events, and fatigue, pyrexia, and anemia predominated (Rannikko et al., 2023). Paired and spatial analyses associated disease control with macrophage activation, interferon-γ, and T-cell-receptor programs, but the most detailed tissue comparison involved a small selected subset. Clever-1 immunostaining and rising serum interferon-γ are exploratory, lack a locked prospective threshold, and may reflect baseline inflammatory competence or downstream activation rather than intratumoral target engagement.

**TABLE 4:** Clinical pharmacology of emerging myeloid checkpoints.

Agent/target	Reported schedule and evidence base	Major safety findings	Dosing challenge	Biomarker limitation
Bexmarilimab/Clever-1 (45)	0.1–10 mg/kg IV every 3 weeks; MATINS, n = 138	Treatment-related events 50%; grade 3–4 8.7%; mainly fatigue, pyrexia, anemia; no DLT or treatment-related death	No MTD and no clear dose–toxicity relation; peripheral or serum changes may not identify tumor biological optimum	Clever-1 staining and serum IFNγ exploratory; small selected paired/spatial subset; no locked cutoff or randomized validation
PY314/TREM2 (46)	10 mg/kg IV plus pembrolizumab 200 mg every 21 days; RCC, n = 17	Treatment-related events 47.1%; grade ≥3 5.9%; fatigue, pyrexia, nausea, infusion reactions	Fixed high dose studied in a small refractory cohort; no demonstrated tumor depletion plateau	No validated TREM2-dependence assay; sparse paired tissue; compensatory TAM states not quantified
MK-4830/LILRB2 (48)	Escalating IV doses every 3 weeks alone or with pembrolizumab; n = 84 in dose escalation	No DLT/MTD; related events 48% monotherapy and 54% combination; grade 3–4 6% and 8%, including hepatic enzymes, fatigue, pneumonitis, hyperglycemia, hypotension	Dose-related engagement did not define a tumor biological optimum; combination toxicity/biology partly inseparable	Exploratory IFNγ signature lacks threshold and prospective validation; intratumoral lineage-specific conversion incomplete
IO-108/LILRB2 (49)	Escalation to 1,800 mg IV every 3 weeks alone or with pembrolizumab; n = 25	Related events 50.0% and 46.2%, all grade 1–2; myalgia/pruritus or pruritus/diarrhea; no DLT	Full blood occupancy at ≥600 mg may encourage escalation without proof of tumor saturation	Peripheral occupancy is not tumor engagement; exploratory tumor-inflammation score and gene changes from small mixed-histology cohorts

Abbreviations: DLT, dose-limiting toxicity; IFN, interferon; IV, intravenous; MTD, maximum tolerated dose; TAM, tumor-associated macrophage; RCC, renal-cell carcinoma.

TREM2 identifies suppressive TAM subsets in several tumors, but depletion does not guarantee restoration of immunity. PY314 was administered at 10 mg/kg with pembrolizumab every 21 days in checkpoint-refractory renal-cell carcinoma. Treatment-related events occurred in 47.1% of 17 patients, with one grade 3 or higher event; fatigue, pyrexia, nausea, and infusion reactions were the most common. One partial response and median progression-free survival of 1.4 months argue against overcoming established checkpoint resistance in this setting (Beckermann et al., 2024). The main translational obstacles are the absence of a validated TREM2-dependent tumor selection assay, limited paired-tissue evidence that the intended subset was depleted, and the possibility that another macrophage population replaced it.

LILRB2 blockade can promote inflammatory macrophage and T-cell functions in preclinical systems (Jones et al., 2025). MK-4830 was given intravenously every 3 weeks as monotherapy or with pembrolizumab; no dose-limiting toxicity occurred and the maximum tolerated dose was not reached. Treatment-related adverse events occurred in 48% of monotherapy and 54% of combination recipients, with grade 3–4 events in 6% and 8%, respectively; fatigue, hepatic-enzyme elevations, pneumonitis, hyperglycemia, and hypotension accounted for the higher-grade related events (Siu et al., 2022). Dose-related target engagement was demonstrated, but the exploratory baseline interferon-γ signature did not provide a validated selection threshold, and combination responses cannot isolate the MK-4830 contribution.

IO-108 was escalated to 1,800 mg every 3 weeks alone or with pembrolizumab. Investigators reported no dose-limiting event; treatment-related events affected 50.0% and 46.2% of patients, respectively, and all were grade 1–2. Myalgia and pruritus were most frequent with monotherapy, while pruritus and diarrhea recurred in the combination cohort. Full peripheral receptor occupancy occurred at doses of at least 600 mg, but blood occupancy is not intratumoral engagement (Taylor et al., 2024). Pharmacodynamic tumor gene-expression changes and baseline tumor-inflammation scores were exploratory in small, histologically mixed cohorts and require spatial lineage assignment, a locked cutoff, and independent validation.

These studies establish feasibility and define manageable early safety profiles, but they do not yet establish a biologically optimal dose. Dose selection should integrate tumor occupancy, conversion of the target-defined lineage, preservation of cDC1/cDC2 function, exposure–response, and toxicity. A plateau in peripheral occupancy should trigger tumor sampling rather than automatic escalation. Randomized expansion should proceed only after a target-dependent baseline population and a reproducible Level 2 assay are established.

## 5 Compensation, mechanism-matched combinations, and disease context

Compensation and redundancy provide the biological through-line that explains why a proximal myeloid effect so often fails to become ecosystem conversion. The relevant unit is a circuit rather than a single cell type: recruitment axes substitute for one another, resident and recruited macrophages differ in survival requirements, and productive interferon signaling creates its own inhibitory feedback. Figure 3 consolidates three recurrent circuits and links each to a specific trial-design response.

**FIGURE 3:**
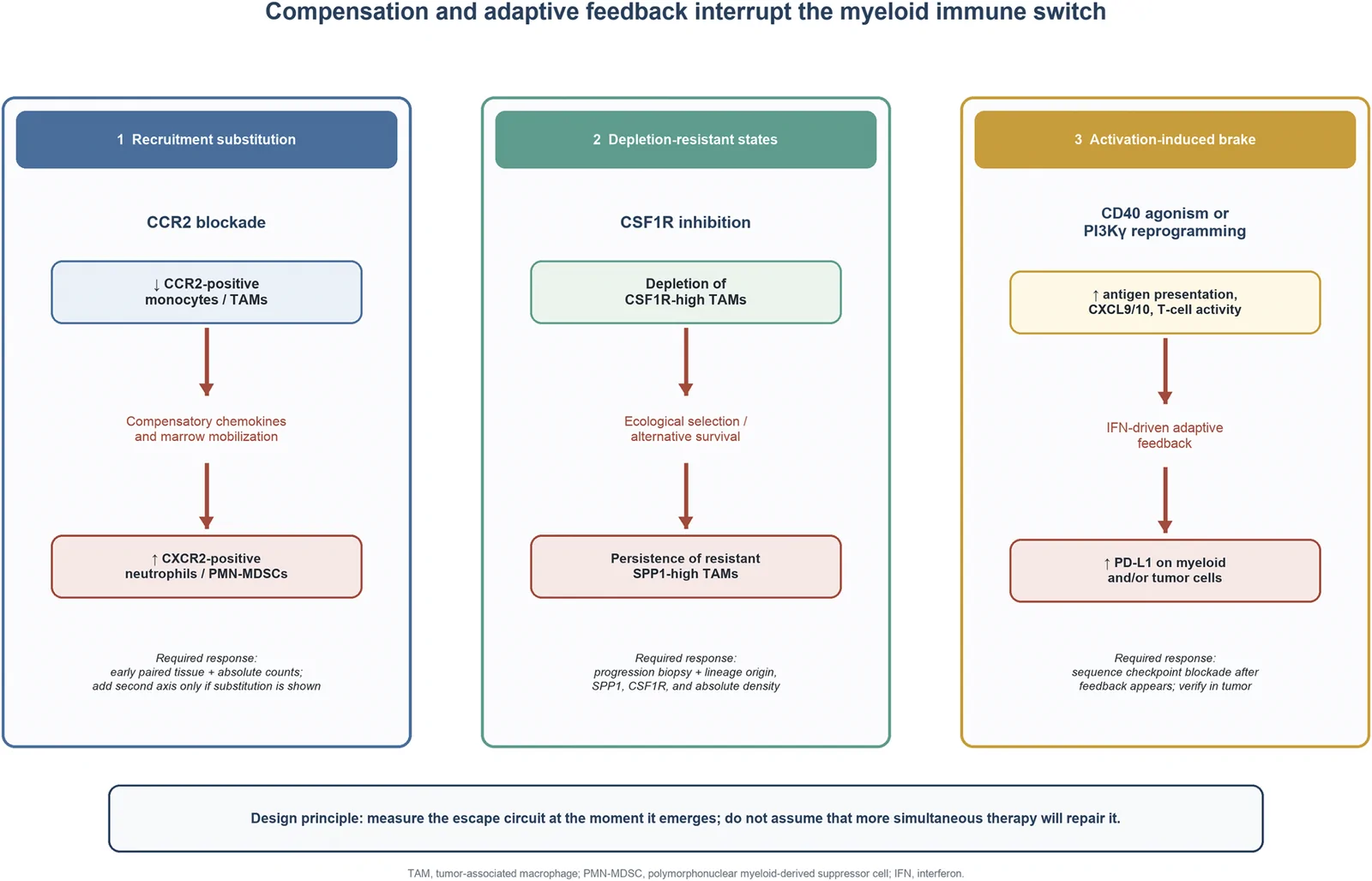
Compensation and adaptive feedback that interrupt myeloid immune conversion. CCR2-positive monocyte/macrophage blockade can be followed by CXCR2-positive neutrophil influx; CSF1R-dependent macrophage depletion can spare or select resistant SPP1-high states; and inflammatory activation through CD40 or PI3Kγ can generate a PD-L1 brake downstream of interferon. Each circuit predicts a distinct response: early paired tissue with absolute lineage counts, progression biopsy and resistant-state profiling, or sequence-aware checkpoint blockade after the feedback signal appears. These mechanisms justify adaptive combinations based on measured escape rather than empiric multi-agent therapy.

First, CCR2 inhibition can reduce monocyte/macrophage influx while increasing tumor-infiltrating CXCR2-positive neutrophils. This substitution was observed in paired PDAC samples and recapitulated in mouse models, where blockade of either CCR2-positive TAMs or CXCR2-positive neutrophils produced compensatory expansion of the alternative subset (Nywening et al., 2018). The implication is not that every patient requires dual blockade. Baseline and early on-treatment tissue should identify which arm is active, and escalation to broader recruitment blockade should require evidence of substitution because combined inhibition increases marrow, infection, and pulmonary risk.

Second, macrophage depletion is selective pressure. In prostate-cancer models, immunosuppressive SPP1-high TAMs expressed relatively low CSF1R and remained largely resistant to anti-CSF1R treatment while other TAM subsets were ablated (Lyu et al., 2025). CSF1R blockade can also remove protective resident macrophages or promote compensatory regulatory-cell programs. Absolute lineage counts and a progression biopsy are therefore necessary to distinguish durable conversion from ecological release of a resistant resident or SPP1-high state.

Third, activation creates adaptive inhibition. Agonistic CD40 stimulation can induce T-cell- and interferon-γ-dependent PD-L1 on tumor-infiltrating monocytes and macrophages (Zippelius et al., 2015). PI3Kγ inhibition can similarly promote inflammatory differentiation while leaving or increasing myeloid PD-L1, creating a rationale for checkpoint blockade only after the activating step and its feedback are demonstrated (Jordanides et al., 2026). The combination partner should therefore be selected by the induced defect and sequenced to the kinetics of that defect, rather than added by default.

Histology constrains all three circuits (Table 5). In PDAC, dense stroma, macrophage and neutrophil recruitment, and limited baseline T-cell access make a resectable window study an efficient test of CD40 sequence, antigen-presenting-cell activation, and stromal remodeling; metastatic PDAC adds greater lesion heterogeneity and less reliable serial tissue. In macrophage-rich triple-negative breast cancer (TNBC), the MARIO-3 paired-biopsy data make PI3Kγ a rational state-conversion hypothesis, but chemotherapy and PD-L1 blockade affect the same readouts, so a backbone-controlled arm is indispensable. Microsatellite-stable colorectal cancer (MSS CRC) has repeatedly exposed the limits of systemic pharmacodynamics, trafficking blockade, CXCR4 recruitment, and CD47 combinations; it is best used as a stringent falsification benchmark after Level 1–2 proof, not as the first efficacy population for an unvalidated mechanism. Tenosynovial giant-cell tumor (TGCT) provides the clearest clinical demonstration of CSF1R dependence, while showing why direct pathway-dependent tumor control is distinct from immune conversion in a carcinoma.

**TABLE 5:** Mechanism, best test-bed, and development priority.

Rank/context	Mechanistic reason to test	Current MICAT position	Next decisive study	Position
1. CSF1R in TGCT	Unusually strong CSF1-pathway dependence and randomized pexidartinib efficacy	Level 4 tumor control without Levels 2–3 immune conversion	Pharmacological benchmark; do not extrapolate to carcinoma reprogramming	Pursue for pathway-dependent disease; restrict immune-switch claims
2. CD47–SIRPα plus HER2 opsonization in gastric/GEJ cancer	Retained tumor antigen supplies selective pro-phagocytic geometry	Partial randomized Level 4 signal; tissue Levels 1–3 incomplete	Confirmatory randomized trial with fresh HER2, tumor phagocytosis, myeloid state, and adaptive propagation	High priority if tumor antigen is freshly confirmed
3. PI3Kγ in macrophage-rich TNBC	Reprogrammable TAMs and paired-biopsy CXCL9/SPP1/ECM signal	Strong regimen-level Levels 2–3; agent-specific Level 1 and Level 4 absent	Backbone ± eganelisib with direct tumor engagement and locked conversion score	High mechanistic priority
4. CD40 in resectable PDAC	Accessible surgical tissue; stromal, cDC, and sequence-dependent biology	Partial Levels 1–3; randomized benefit unresolved	Window trial with lead-in, cDC1/cDC2 function, stroma, TCR, and component control	Prioritize sequence-aware tissue-rich studies
5. CCR2/CXCR2 in PDAC	Active monocyte/neutrophil recruitment but demonstrable substitution	Mostly Levels 1–2	Adaptive or dual-axis design only after on-treatment substitution is shown	Narrow to ligand/lineage-enriched tumors
6. MSS CRC	Redundant myeloid/stromal resistance repeatedly defeats partial pharmacology	Recurrent failure before Level 3–4	Use after Level 2 as a stringent falsification cohort	Failure benchmark, not first efficacy test-bed
7. Clever-1, LILRB2, TREM2 across solid tumors	Target-defined suppressive subsets may be actionable	Early Level 1 and partial Level 2–3 signals	Histology-specific selection, tumor occupancy, locked lineage assay, then randomized expansion	Biomarker-development priority; no broad efficacy claim

Abbreviations: cDC, conventional dendritic cell; ECM, extracellular matrix; GEJ, gastroesophageal junction; MICAT, myeloid immune conversion audit trail; MSS CRC, microsatellite-stable colorectal cancer; PDAC, pancreatic ductal adenocarcinoma; TCR, T-cell receptor; TGCT, tenosynovial giant-cell tumor; TAM, tumor-associated macrophage; TNBC, triple-negative breast cancer.

Sequence should follow the expected kinetics. CD40 priming can precede antigen release when dendritic-cell licensing is the intended first step. A tumor-opsonizing antibody should be active when CD47–SIRPα blockade begins. PD-1/PD-L1 blockade is rational after antigen presentation, CXCL9/CXCL10 induction, or an interferon-linked PD-L1 increase is shown. At progression, tissue should test whether the original target was lost, the intended population recovered, or a new suppressive program became dominant. Adaptive, mechanism-triggered combinations yield more interpretable evidence than fixed multi-agent regimens in unselected populations.

## 6 Translational proof of an immune-state switch

### 6.1 Tissue sampling, timing, and analytical discipline

Tissue procurement should follow standardized acquisition, processing, and chain-of-custody procedures (Gastman et al., 2020). Figure 4 gives a worked randomized, biopsy-embedded design: patients are assigned to a common backbone or the same backbone plus the myeloid agent; fresh baseline tissue establishes target availability; an acute 4–24-h blood sample and optional 24–72-h tumor sample assess pharmacokinetics and Level 1; a day 7–14 lead-in biopsy tests Level 2 before later partner effects dominate; a week 3–8 specimen or surgical sample tests Level 3; and response, pathological endpoints, circulating tumor DNA, progression-free survival, and overall survival support Level 4. Timing must be adapted to target turnover and agent pharmacology.

**FIGURE 4:**
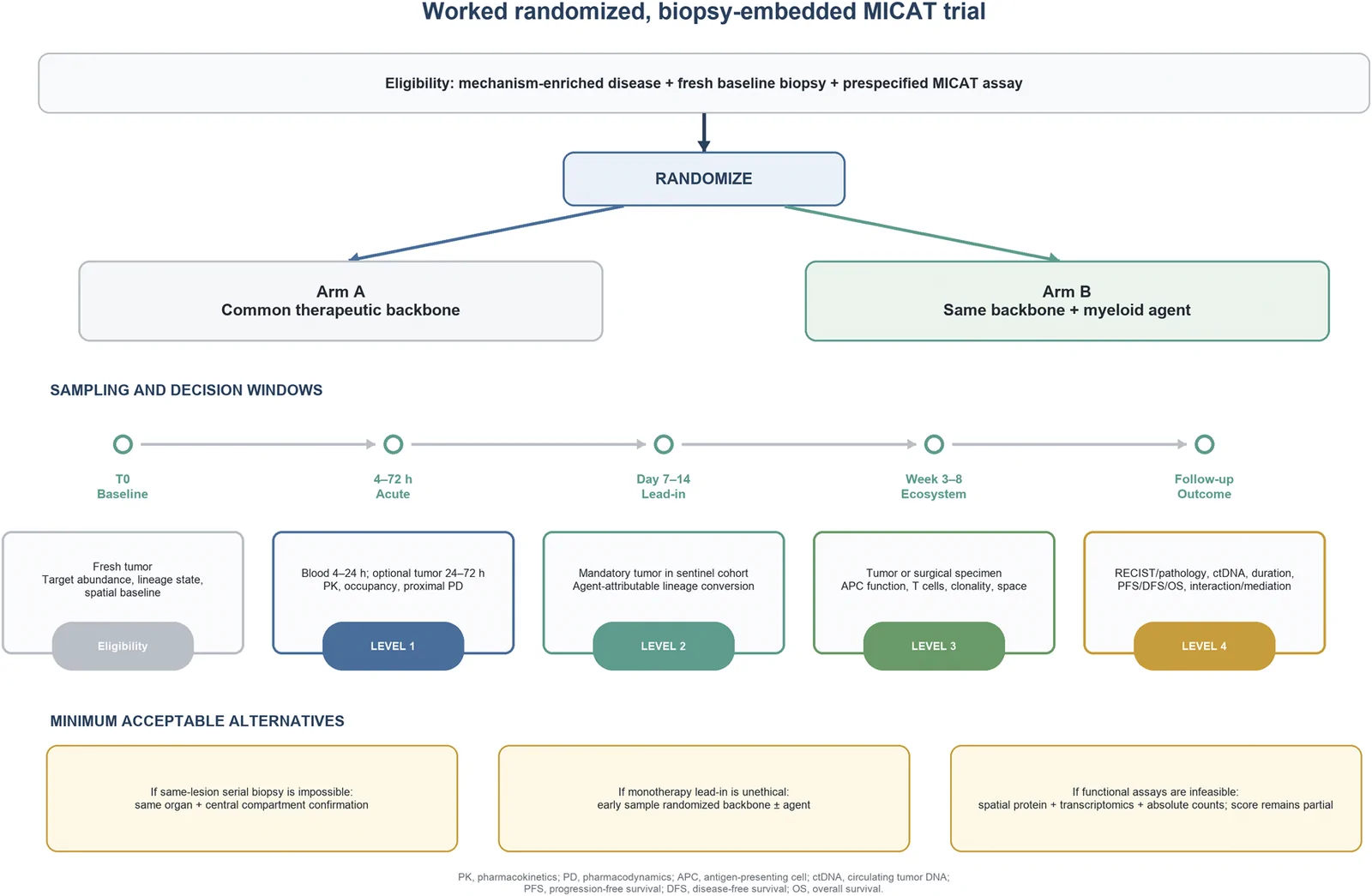
Worked design for a randomized, biopsy-embedded MICAT trial. The experimental arm adds the myeloid drug to an otherwise identical control backbone. Pretreatment tissue confirms target availability; acute blood and optional tumor sampling test exposure and engagement; a day 7–14 lead-in sample isolates target-lineage conversion; a week 3–8 biopsy or surgical specimen tests ecosystem propagation; and clinical endpoints establish randomized attribution. A prespecified sentinel biopsy cohort and same-organ paired tissue are minimum alternatives when the full schedule is impractical. MICAT, Myeloid Immune Conversion Audit Trail; PK, pharmacokinetics; PD, pharmacodynamics; ctDNA, circulating tumor DNA; ORR, objective response rate; PFS, progression-free survival; OS, overall survival.

Same-lesion serial sampling reduces anatomical confounding when feasible, whereas multiregion baseline sampling addresses heterogeneity. Previously irradiated lesions should be avoided and cores allocated prospectively to fixed-tissue, fresh-tissue, and functional analyses. Failed post-treatment biopsies must be reported because rapidly progressing or clinically unstable patients are less likely to provide evaluable tissue.

The complete package is not practical in every solid tumor. The minimum acceptable alternative is one mandatory on-treatment tumor sample in a prespecified sentinel cohort, paired with a fresh baseline sample from the same lesion or, when that is impossible, the same organ and centrally confirmed compartment. If a monotherapy lead-in is unethical, early sampling in a randomized backbone-versus-backbone-plus-agent design should occur before response or cumulative partner effects dominate. Validated imaging of tumor delivery, plasma pharmacokinetics, circulating proximal pharmacodynamics, and circulating tumor DNA can support Levels 1 and 4 but cannot substitute for lineage-resolved tissue at Levels 2–3. If a functional assay is infeasible, concordant spatial protein and transcriptomic evidence with absolute cell counts is acceptable only as partial Level 2–3 evidence. Missing-tissue rules and the maximum attainable MICAT score should be specified before enrollment.

Cells, image regions, and transcriptomic spots are nested observations, not independent patient replicates. Analyses should use patient-level summaries or mixed-effects models, control multiplicity, and report the biomarker-evaluable population separately from the intention-to-treat population. The state score, assay, threshold, tissue compartment, and success criterion should be specified before outcomes are examined. A *post hoc* signature optimized on responders is discovery, not validation.

### 6.2 Measuring conversion: composition, function, and space

A macrophage-directed study should report both absolute densities and proportions. A higher HLA-DR-positive/HLA-DR-negative ratio may reflect activation, selective depletion, or recruitment of a new population. Single-cell RNA sequencing or CITE-seq can define state, while multiplex immunofluorescence or imaging mass cytometry provides absolute density and neighborhood information. An orthogonal functional assay—phagocytosis, antigen uptake, cross-presentation, or cytokine production—strengthens causal interpretation.

Useful state domains include a suppressive/remodeling module comprising SPP1, MRC1, ARG1, IL10, transforming-growth-factor-β-related and extracellular-matrix genes, and an antigen-presentation/inflammatory module comprising HLA class II, CD74, CIITA, CD86, IL12B, CXCL9, CXCL10, and interferon-response genes. The CXCL9:SPP1 axis is a promising continuous summary but is not a prospectively validated pharmacodynamic or predictive biomarker (Bill et al., 2023). Its implementation faces several practical barriers: the ratio is compositional and can change through depletion of one population rather than conversion of the same cells; bulk, single-cell, and spatial platforms measure different compartments and dynamic ranges; interferon exposure, corticosteroids, infection, and biopsy timing alter CXCL9; SPP1 is produced by non-macrophage stromal and tumor cells; small cores undersample spatially segregated niches; and no cross-platform normalization method, locked cutoff, reference standard, or prospective decision rule exists. A clinically usable assay therefore requires lineage assignment, absolute macrophage density, batch-locked processing, same-compartment sampling, prespecified timing, analytical reproducibility, and prospective validation across independent cohorts.

Evidence should also show propagation beyond macrophages: mature cDC1 and cDC2 states, cross-presentation or an appropriate surrogate, activated or cytotoxic CD8 cells, IFNG/GZMB/PRF1, tumor-reactive T-cell clonotypes, a favorable effector-to-regulatory ratio, or organized tertiary lymphoid structures. A machine-learning analysis of neoadjuvant CD40 therapy in PDAC linked the localization and function of CD44-positive CD4 T cells to disease-free survival (Blise et al., 2024). Conversely, CXCR4 inhibition plus PD-1 blockade increased T-cell recruitment and macrophage–T-cell proximity but also enriched CD206-high macrophages and produced little clinical activity (Bever et al., 2025), showing that infiltration and proximity alone do not satisfy ecosystem-level conversion.

Histologic tumor budding can provide a contextual spatial endpoint but should not enter an immune-conversion composite without paired immune measures and prospective validation; in the authors' exploratory cohort of colorectal liver metastases, immune markers were unavailable and the prognostic association was limited to the two-year outcome in synchronous disease (Pe et al., 2026).

### 6.3 Baseline enrichment and host-extrinsic modifiers

Baseline target expression is often prognostic rather than predictive. Predictive value is most convincingly established by a treatment-by-biomarker interaction in randomized data. Enrichment should therefore combine target availability with pathway dependence: a CSF1R trial needs a CSF1-dependent suppressive population and preserved dendritic-cell capacity; a CXCR2 trial needs an active ligand/recruitment program; a PI3Kγ trial needs abundant reprogrammable macrophages; a CD40 trial needs competent antigen-presenting cells; and a CD47 trial needs retained tumor antigen and a pro-phagocytic partner.

Microbiome-mediated checkpoint resistance in MSI-high/mismatch-repair-deficient colorectal cancer has been reviewed in detail (Marjanovic et al., 2026), but in trials of myeloid-directed agents microbiome measurements should remain prespecified host-level covariates because taxonomic associations cannot establish intratumoral engagement or conversion.

### 6.4 Trial designs that can establish causal contribution

For clinical attribution, randomized add-on trials should compare a standard backbone with the same backbone plus the myeloid modulator. When two experimental partners are introduced, a three-arm or factorial design may be required. Single-arm triplets can show feasibility, tissue pharmacodynamics, and a response signal but cannot identify which component caused benefit. Figure 4 operationalizes this design and ties every sampling point to a MICAT decision.

Dose finding should prioritize the biologically optimal dose. Intratumoral receptor occupancy, state conversion, preservation of beneficial myeloid and dendritic populations, toxicity, and exposure–response should be integrated. Higher continuous exposure can deepen depletion while impairing antigen presentation or selecting resistant states. Randomized dose comparison is preferable when pharmacodynamic saturation occurs below the maximum tolerated dose.

An on-treatment specimen may be classified as provisionally positive for biological conversion when intratumoral target engagement and a prespecified lineage-specific state change are accompanied by concordant downstream changes in at least two orthogonal domains, one of which measures adaptive effector activation. Thresholds should be calibrated, locked, and externally validated. This pharmacodynamic composite is not a surrogate of benefit; Level 4 still requires randomized clinical attribution, while interaction or mediation analyses test the composite’s clinical relevance.

## 7 Therapeutic window, safety, and emerging platforms

### 7.1 Safety is part of switch biology

Mechanism-specific toxicities are biological readouts of normal-tissue target engagement and therefore belong in the pharmacodynamic model. A fall in circulating neutrophils may confirm CXCR2 inhibition while defining the limiting exposure. Cytokines after CD40 agonism may indicate pathway activation but do not establish tumor localization. Anemia after CD47 blockade confirms normal-cell engagement, not desirable pharmacology. Trials should prespecify stop–go rules that integrate tissue conversion with toxicity. If the biologically active exposure is reached without ecosystem propagation, dose escalation is unlikely to repair the mechanism and may only narrow the therapeutic window.

Localized, conditional, bispecific, or Fc-tuned constructs trade systemic toxicity against tumor exposure and activation geometry; their evaluation therefore requires paired pharmacokinetic, pharmacodynamic, and functional tissue readouts. The engineering objective is sufficient, appropriately timed activity in the cellular neighborhood needed for tumor rejection.

### 7.2 Forward platforms under the same audit trail

New platforms change delivery and cellular geometry but do not remove the need for causal evidence (Table 6). In the first-in-human CT-0508 study, autologous anti-HER2 CAR-macrophages were manufactured and administered without lymphodepletion; serial biopsies confirmed tumor trafficking and microenvironment remodeling with CD8 expansion, but 4 of 9 patients with HER2 3+ tumors had stable disease and no objective responses were reported in the interim analysis (Reiss et al., 2025). This supports Level 1 and partial Levels 2–3, while persistence, antigen escape, manufacturing variability, and randomized attribution remain unresolved.

**TABLE 6:** Emerging platforms mapped to MICAT.

Platform	Current evidence	Required Level 1–2 evidence	Required Level 3–4 evidence	Principal translational risk
*Ex vivo* CAR-macrophages/CT-0508 (59)	Human phase I manufacturing, safety, tumor trafficking, partial remodeling	Tumor CAR-M abundance/persistence, antigen engagement, phagocytosis, stable functional state	Adaptive propagation, antigen-spreading assay, randomized component contribution	Manufacturing variability, limited persistence, antigen escape, tumor entry
*In vivo* mRNA/LNP macrophage programming (Gu et al., 2025)	Preclinical CAR expression, state change, ecosystem remodeling	Human biodistribution, lineage-specific transfection, expression duration, repeat-dose PK/PD	Durable T-cell propagation and randomized benefit against matched backbone	Liver/spleen uptake, off-lineage transfection, innate toxicity, transient expression
Bispecific/trispecific myeloid engagers (Zhao et al., 2026)	Preclinical tumor-antigen targeting plus pro-phagocytic activation/checkpoint blockade	Tumor colocalization, simultaneous arm engagement, tumor-selective phagocytosis	Antigen transfer, T-cell activation, randomized benefit	Off-tumor phagocytosis, cytokines, structural geometry, antigen heterogeneity
AI-assisted spatial scoring (Blise et al., 2024; Tarhini and El Naqa, 2025)	Retrospective and exploratory spatial prediction	Locked cell/compartment definitions, analytical reproducibility, external validation	Prospective treatment interaction and mediation, randomized validation of clinical utility	Domain shift, sampling bias, opaque features, overfitting, platform dependence

Abbreviations: AI, artificial intelligence; CAR, chimeric antigen receptor; LNP, lipid nanoparticle; MICAT, myeloid immune conversion audit trail; PD, pharmacodynamics; PK, pharmacokinetics.

*In vivo* mRNA/lipid-nanoparticle programming could avoid individualized *ex vivo* manufacturing and permit repeat dosing. Preclinical intraperitoneal CAR-mRNA nanoparticles generated proinflammatory CAR-macrophages, remodeled the immune ecosystem, and induced PD-L1 as a measurable feedback program (Gu et al., 2025). Logic-gated macrophage engagers that combine a tumor-antigen arm, pro-phagocytic activation, and SIRPα blockade have likewise produced antigen-dependent activity in mouse solid-tumor models, including expression from mRNA nanoparticles (Zhao et al., 2026). Both approaches still require human biodistribution, lineage specificity, duration of expression, off-tumor phagocytosis, cytokine safety, and component control.

Artificial-intelligence-assisted spatial scoring can scale neighborhood analysis from multiplex assays or routine histology, but retrospective prognostic performance is not a Level 2 or 3 pharmacodynamic result. Models must use locked cell definitions and compartments, be tested for scanner, stain, center, and ancestry shift, and undergo external and prospective validation before serving as an enrichment or response assay (Blise et al., 2024; Tarhini and El Naqa, 2025). Explainable features should be linked to measured cell states rather than used as opaque surrogates. MICAT therefore treats these platforms as tools for evidence generation, not evidence shortcuts.

## 8 Discussion and priorities for clinical development

The MICAT matrix shows that the field’s limiting step is not a shortage of targets. Most programs establish systemic exposure or a proximal signal, fewer demonstrate lineage-resolved conversion, and still fewer show that the change propagates through dendritic-cell and T-cell function. Randomized benefit attributable to a myeloid agent remains exceptional. Compensation is the most coherent explanation: a depleted or blocked population is replaced, a resistant state persists, or successful activation induces a new inhibitory feedback.

### 8.1 Ranked development priorities

The nearest direct Level 4 evidence is CSF1R inhibition in TGCT, where ENLIVEN demonstrated randomized tumor responses. This should be pursued as a pathway-dependence model and a pharmacological benchmark, while explicitly avoiding the claim that it validates immune conversion in carcinomas. Among carcinoma strategies, tumor-selective CD47–SIRPα blockade paired with retained HER2 opsonization in gastric or gastroesophageal cancer is closest to a testable Level 4 hypothesis; the ASPEN-06 fresh-biopsy signal remains exploratory and conference-reported, so confirmatory randomized evidence and paired phagocytosis/ecosystem sampling are required before clinical priority is assigned.

PI3Kγ inhibition in macrophage-rich TNBC has the strongest current Levels 2–3 signal because paired biopsies show macrophage, interferon, and extracellular-matrix remodeling. It should move to a backbone-controlled trial with direct intratumoral target engagement and a locked conversion score. Sequence-aware CD40 agonism should be prioritized in resectable or window-of-opportunity PDAC, where surgery supplies tissue and stromal, cDC, and T-cell effects can be measured before metastatic heterogeneity obscures them. Recruitment blockade should proceed only in tumors with an active ligand/lineage program and an explicit plan to detect CCR2/CXCR2 substitution. Clever-1 and LILRB2 merit biomarker-development cohorts; TREM2 depletion should not advance in checkpoint-refractory renal-cell carcinoma without evidence that a TREM2-dependent population is present and not replaced.

### 8.2 Mechanisms to narrow or retire

Several generic development strategies should be retired. These include unselected CSF1R inhibition added empirically to PD-1/PD-L1 blockade; single-axis CCR2 or CXCR2 inhibition in late-line, unselected tumors without an on-treatment compensation assay; active-Fc pan-CD47 therapy without tumor-selective opsonization and hematologic-risk mitigation; and efficacy claims from multi-agent single-arm regimens that lack component control. Retirement applies to the broad strategy, not to a biomarker-defined exception supported by new tissue evidence.

MSS CRC should be used deliberately as a failure benchmark after a mechanism reaches Level 2, because its redundant myeloid and stromal barriers expose weak or incomplete conversion. It should not be the first proof-of-concept efficacy setting for a drug supported only by blood pharmacodynamics. The most favorable settings are those with a demonstrable dependency, retained tumor antigen for selective opsonization, abundant but reprogrammable macrophages, or a resectable window that provides high-quality tissue.

The practical go/no-go rule is stringent. Stop dose escalation when tumor engagement is saturated but Level 2 is absent; redesign the combination when Level 2 occurs without Level 3; and do not call a clinical switch until randomized benefit completes the chain. Using negative trials in this way turns failure into mechanism-specific information and prevents repeated testing of pharmacologically active but ecologically incomplete interventions.
